# Delayed Fever and Acute Kidney Injury in Patients with Urinary Tract Infection

**DOI:** 10.3390/jcm9113486

**Published:** 2020-10-28

**Authors:** Kun-Lin Lu, Chih-Yen Hsiao, Chao-Yi Wu, Chieh-Li Yen, Chung-Ying Tsai, Chang-Chyi Jenq, Hsing-Lin Lin, Yu-Tung Huang, Huang-Yu Yang

**Affiliations:** 1 Department of Medical Education, Chang Gung Memorial Hospital at Linkou, Taoyuan 333, Taiwan; johnlu710999@gmail.com; 2 Division of Nephrology, Department of Internal Medicine, Ditmanson Medical Foundation Chia-Yi Christian Hospital, Chia-Yi 600, Taiwan; ebct2@yahoo.com.tw; 3 Department of Hospital and Health Care Administration, Chia Nan University of Pharmacy and Science, Tainan 71710, Taiwan; 4 Division of Allergy, Asthma, and Rheumatology, Department of Pediatrics, Chang Gung Memorial Hospital, Chang Gung University College of Medicine, Taoyuan 333, Taiwan; joywucgu@hotmail.com; 5 Kidney Research Institute, Nephrology Department, Chang Gung Memorial Hospital, College of Medicine, Chang Gung University, Taoyuan 333, Taiwan; b9102087@yahoo.com.tw (C.-L.Y.); cytsai0616@cgmh.org.tw (C.-Y.T.); edward.jenq@gmail.com (C.-C.J.); 6 Division of Critical Care Surgery, Department of Critical Care Medicine, Veterans General Hospital, Kaohsiung 813, Taiwan; hsinglin2002@yahoo.com.tw; 7 Center for Big Data Analytics and Statistics, Chang Gung Memorial Hospital, Taoyuan 333, Taiwan; anton.huang@gmail.com; 8 Department of Health Policy and Management, Johns Hopkins Bloomberg School of Public Health, Baltimore, MD 21205, USA

**Keywords:** urinary tract infection, acute kidney injury, afebrile urinary tract infection, delayed fever, baseline renal function, length of hospital stay, aging, leukocytosis

## Abstract

The presence of fever has long been a warning sign of severe urinary tract infection (UTI). However, we previously identified that inpatients with afebrile UTI had an increased risk of developing acute kidney injury (AKI). After expanding this cohort, 1132 inpatients with UTI diagnosed between January 2006 and April 2019 were analyzed. Overall, 159 (14%) of these patients developed AKI; bacteremia, urolithiasis, septic shock, hypertension, lower baseline renal function, marked leukocytosis, and the absence of fever were independently linked to AKI. When we further studied the cohort of inpatients with fever during hospitalization, we identified a group of “delayed fever” UTI inpatients who did not have fever as their initial presentation. Compared to patients presenting with fever at the emergency department, patients with delayed fever tended to be younger and have less frequent infection with *Escherichia coli*, more frequent AKI, upper tract infection, and a longer hospital stay. Despite the initial absence of fever, these patients demonstrated larger extents of elevations in both serum white blood cell counts and C-reactive protein levels. In short, besides UTI patients with lower baseline renal function that remain afebrile during their hospital stay, clinical awareness of the increased incidence of AKI in younger patients with “delayed fever” should also be noted.

## 1. Introduction

As one of the most common infectious entities worldwide, urinary tract infection (UTI) is a vital global health burden [1,2]. For example, it was reported that UTI accounted for nearly two million visits to the emergency department per year in the United States alone [3]. Acute UTIs include acute cystitis and acute pyelonephritis (APN) [4]. APN and bacteremia are more closely linked to life-threatening illnesses, as well as complications such as renal scarring and impaired renal function [5,6,7], and they therefore require timely diagnosis and treatment. In clinical scenarios, fever in patients with UTI raises concerns about the presence of either APN or bacteremia [8,9]. Moreover, elevated body temperature also reduces renal blood flow and glomerular filtration rates (GFR), leading to an increased plasma level of creatinine [10,11]. Therefore, it is straightforward for physicians to believe that febrile UTI patients may be more prone to subsequent complications such as acute kidney injury (AKI) than are UTI patients without fever. However, our previous investigation revealed that the UTI patients remaining afebrile during hospitalization were significantly more susceptible to developing AKI [6]. Since, to our knowledge, there has not been any literature focusing on exploring the cause of this intriguing phenomenon, we sought to untangle the possible contributing factors, and meanwhile managed to point out room for improvement in the clinical care of UTI patients. Herein, we expanded the previous cohort of UTI patients and managed to identify how the presence of fever interacted with various clinical conditions and outcomes.

## 2. Materials and Methods

### 2.1. Ethics Statement

This retrospective observational study complied with the guidelines of the Declaration of Helsinki and was approved by the Medical Ethics Committee of Chia-Yi Christian Hospital, a tertiary referral center located in the southwestern region of Taiwan. Approval from the Institutional Review Board of Chia-Yi Christian Hospital was also obtained (Approval # CYCH-IRB-2019061), but without specific informed consent from patients. All data were analyzed anonymously (by delinking identifying information from the main data sets) and made available only to investigators. The Institutional Review Board of Chia-Yi Christian Hospital specifically waived the need for consent for these studies. All primary data were collected according to procedures outlined in the epidemiology guidelines.

### 2.2. Study Conduct

This retrospective study was conducted in a tertiary referral center with 1077 acute care beds, which serves approximately 4110 outpatients and 260 emergency patients daily. The authors had full access to the results and vouch for the completeness and accuracy of the data and analysis.

### 2.3. Study Population

From January 2006 to April 2019, patients that were admitted for UTI with laboratory data at baseline and during hospitalization, and who received one or more imaging studies (including ultrasonography, intravenous urography, or computed tomography) during hospitalization were included. The clinical data of 1132 hospitalized patients with baseline creatinine values diagnosed with UTI in the Chia-Yi Christian Hospital were used. The criteria for the diagnosis of UTI were being symptomatic, including pain on urination, lumbar pain, or fever with leukocyturia, and a urine bacterial isolation of more than 10^4^ colony forming units (CFU)/mL. All patients underwent imaging studies, such as ultrasonography, intravenous urography or computed tomography. Patients with concurrent infections other than UTI or receiving chronic dialysis therapy (i.e., regular dialysis therapy for more than 3 months) before the UTI episode were excluded from this study. Hospital Course Inpatients were assessed by standard laboratory and diagnostic procedures. Clinical data, including age, gender, diabetes mellitus (DM), hypertension, coronary artery disease (CAD), congestive heart failure (CHF), cerebrovascular disease, laboratory results (white blood cell (WBC) count, platelet count, serum creatinine, and estimated glomerular filtration rate (eGFR) at baseline and after hospitalization), and causative microorganisms and antimicrobial resistance patterns, were collected using a standard form for further analysis. Admitted patients were treated with antibiotics based on the standard protocol. The initial regimens of empiric antibiotic therapy were parenteral first-generation cephalosporin plus amino-glycoside (if no impaired renal function was found), parenteral second-generation cephalosporin or parenteral fluoroquinolones to treat common UTI pathogens for patients with stable hemodynamic conditions. Parenteral empiric antibiotic therapy according to previous culture results and antimicrobial susceptibility was prescribed for patients with recurrent UTI. Specific antibiotic therapy was administered according to the culture results and antimicrobial susceptibility during hospitalization. The four main vital signs, including heart rate, respiration rate, blood pressure and temperature, were routinely monitored. The patients were divided into two groups based on the presence or absence of fever during hospitalization.

### 2.4. Major Outcomes and Definitions

AKI was defined as an increase in serum creatinine to ≥2.0 times the baseline value, according to the Kidney Disease: Improving Global Outcomes Clinical Practice Guideline criteria for serum creatinine values for AKI stages 2 and 3 [12]. eGFR was determined according to the Chronic Kidney Disease Epidemiology Collaboration (CKD-EPI) creatinine equation [13]. The baseline levels of serum creatinine were obtained at the regular follow-up-date about 3 months before admission. The diagnosis of DM was made according to the American Diabetes Association and the World Health Organization criteria [14]. Upper UTI was an infection of the kidney or ureter; lower UTI included cystitis, urethritis, and prostatitis. Bacteremia was defined as an invasion of the bloodstream by bacteria and was confirmed by blood culture. Fever was defined as a temperature above 38.3 °C (101 °F) [15,16]. Afebrile UTI was defined as UTI patients who did not have a temperature above 38.3 °C (101 °F). Septic shock was defined as sepsis with hypotension (systolic blood pressure less than 90 mmHg or a fall in systolic blood pressure > 40 mmHg) lasting for at least 1 h despite adequate fluid resuscitation [17]. Multiple drug resistance (MDR) was defined as the non-susceptibility of isolates to at least one agent in each of any three or more antimicrobial categories [18].

### 2.5. Study Design of Sub-Group Analysis

First, all UTI patients recruited were divided into two groups for comparison based on the presence or absence of fever, AKI or bacteremia during the hospital stay. Second, for UTI patients that suffered from fever during hospitalization, we further separated them according to the presence or absence of fever at ED. In order to identify the UTI populations that were more susceptible to fever, AKI and bacteremia, we reviewed medical charts and included demographic data of gender, age, co-morbidities (i.e., diabetes mellitus, hypertension, CHF, CAD, stroke, and malignancy), baseline renal function, vital signs at admission, as well as indwelling urinary catheter. To look into what the presence of fever, AKI or bacteremia along with UTI may be subsequently linked to, the diagnosis of upper or lower UTI, and the presence of bacteremia or septic shock during hospitalization, were also reviewed from medical charts for further analysis.

### 2.6. Statistical Analysis

The patients were grouped into two categories: those with and those without fever during hospitalization. The data were analyzed using Student’s *t*-test or chi-square test depending on the types of variables. Continuous variables are expressed as mean ± standard deviation (SD), and categorical variables are expressed as number (and percentage). Only variables significant at the 0.15 level in the univariate analysis were selected for consecutive multivariate analyses. Multivariate logistic regression analyses were performed to identify factors associated with fever, bacteremia, septic shock and AKI post-admission. In general, a *p*-value below 0.05 was considered significant. In order to address the multiple testing problem, we applied Holm–Bonferroni Correction in the multivariate analysis, reporting both the *p*-values and each of their significance levels (α). Statistical analyses were performed with the software SPSS 17.0 (International Business Machines Corp., Armonk, NY, USA).

## 3. Results

### 3.1. Demographic and Clinical Characteristics

The demographic and clinical characteristics of the 1132 UTI patients recruited are shown in Table 1. The mean age of these patients was 66 ± 17 years, and 297 (26.2%) of them were male. Hospital stays lasted 9 ± 5 days, and there were 540 (47.7%) patients with bacteremia, 218 patients (19.3%) with septic shock and 159 patients (14.0%) with AKI during hospitalization. As compared to patients with afebrile UTI, patients with febrile UTI had a significantly younger age (64 ± 18 versus 71 ± 16 years, *p* < 0.001), higher baseline eGFR (80.0 ± 28.6 versus 70.4 ± 31.1 mL/min/1.73m^2^, *p* < 0.001), were more likely to be female (75.7% versus 70.3%, *p* = 0.045), showed correlation with bacteremia (57.0% versus 31.2%, *p* < 0.001), septic shock (21.7% versus 15.0%, *p* = 0.006) and *Escherichia coli (E.coli)* isolates (83.3% versus 72.2%, *p* < 0.001), had lower incidences of comorbidities (hypertension (50.3% versus 61.2%, *p* < 0.001) and stroke (15.9% versus 29.0%, *p* < 0.001)), were less in need of an indwelling Foley catheter (4.1% versus 10.1%, *p* < 0.001), and had less AKI (12.0% versus 17.7%, *p* = 0.008). There was no significant difference noted in the frequency of MDR isolates, length of hospital stay, or mortality. 

### 3.2. Risk Factors Related to Acute Kidney Injury in Hospitalized Urinary Tract Infection (UTI) Patients

Of all patients hospitalized due to UTI, those who developed AKI had higher ages, longer hospital stays, higher WBC counts and serum creatinine concentrations on admission, lower platelet counts, higher prevalences of DM, hypertension, CHF, stroke, urolithiasis, bacteremia and septic shock, more *Proteus* species isolates and fewer *E. coli* isolates than patients without AKI (see Table 2). Again, no significant difference in the frequency of MDR isolates or mortality was found. Multivariate logistic regression analysis revealed that hypertension (OR 1.859, 95% CI 1.202–2.874, *p* = 0.005), bacteremia (OR 1.906, 95% CI 1.262–2.878, *p* = 0.002), septic shock (OR 4.417, 95% CI 2.948–6.618, *p* < 0.001), urolithiasis (OR 2.818, 95% CI 1.845–4.303, *p* < 0.001) and higher WBC counts (OR 1.050, 95% CI 1.020–1.080, *p* = 0.001) were independently associated with an increased risk for AKI in patients with UTI. Conversely, high fever (OR 0.521, 95% CI 0.346–0.784, *p* = 0.002) was independently associated with a decreased risk of AKI in patients with UTI (see Table 3). 

### 3.3. Risk Factors Related to Bacteremia in Hospitalized UTI

Patients with bacteremia had more comorbidities (DM and hypertension), lower incidences of stroke comorbidity, longer hospital stays, higher serum creatinine concentrations on admission, lower platelet counts, higher prevalences of DM, hypertension, fever, urolithiasis, septic shock and AKI, and more MDR bacteria isolated than patients without bacteremia (see Table 4). Multivariate logistic regression analysis revealed that DM (OR 1.604, 95% CI 1.223–2.104, *p* = 0.001), fever (OR 3.057, 95% CI 2.306–4.052, *p* < 0.001), septic shock (OR 1.746, 95% CI 1.241–2.456, *p* = 0.001), AKI (OR 1.832, 95% CI 1.226–2.738, *p* = 0.003) and *E. Coli* isolates (OR 1.976, 95% CI 1.976 1.414–2.760, *p* < 0.001) were independently associated with an increased risk for bacteremia in patients with UTI. Conversely, a lower platelet count (OR 0.997, 95% CI 0.995–0.998, *p* < 0.001) was independently associated with a decreased risk for bacteremia in patients with UTI (see Table 5).

### 3.4. Subgroup Analysis for Febrile UTI Cases

As for the 725 patients with documented fever, 714 had their vital signs recorded at the emergency department (ED) upon arrival. These febrile UTI patients were divided into two groups according to the presence or absence of fever at ED. We compared the characteristics of these two groups of patients as shown in Table 6. Among patients with febrile UTI, the presence of fever initially at ED was surprisingly found to be related to older age (64.7 ± 18.1 versus 61.5 ± 17.2 years, *p* = 0.028), less upper urinary tract infection (47.6% versus 59.5%, *p* = 0.004), less subsequent AKI (9.3% versus 17.6%, *p* = 0.001), as well as a shorter hospital stay (9.0 ± 4.4 versus 10.1 ± 5.3 days, *p* = 0.002). Moreover, both lower serum WBCs (13.1 ± 5.8 versus 14.4 ± 6.4 10^3^/μL, *p* = 0.008) and CRP levels (10.6 ± 8.6 versus 12.6 ± 9.8mg/L, *p* = 0.029) were also noted in febrile UTI patients with an initial presentation of fever at ED. Besides these differences, there was no other significantly different factor recorded distinguishing these two groups of patients. Multivariate logistic regression analysis of febrile UTI patients showed that an initial clinical presentation of fever at ED was possibly related to elder age (OR 1.02, 95% CI 1.00–1.03, *p* = 0.010), higher baseline renal function (OR 1.01, 95% CI 1.00–1.02, *p* = 0.022) and more frequent *E. coli* infection (OR 1.58, 95% CI 1.01–2.46, *p* = 0.046), and was significantly linked to less frequent AKI (OR 0.43, 95% CI 0.26–0.72, *p* = 0.001), as shown in Table 7.

## 4. Discussion

Fever has always been one of the most indicative clinical presentations for severe infection in clinical scenarios [8,15]. As an evolutionarily conserved host response to infection, fever is thought to be protective against infection by directly inhibiting the invading microbes, in addition to activating the host’s immune system [19,20]. It is known that enhanced immunologic responses are observed in the setting of elevated temperature within the physiologic range [21], whereas hypothermia independently predicts hospital mortality in septic patients [22]. Since UTI is one of the common causes of fever, as well as subsequent complications including AKI and urosepsis, whether fever per se plays a role in predicting the clinical outcomes of UTI remains to be clarified. In addition, if the presence of fever in UTI patients does link to poor outcomes despite its protective effect, whether this could be purely attributed to its role in indicating more severe ongoing infections, or if it caught clinical attention and thus led to more adequate, timely treatment, was largely unknown.

The average age of our cohort was 66±17 years old, and therefore the results may reflect more the conditions of the elderly. In the elderly, the incidence of UTI is increased by multiple factors, including the elevated incidences of immunity recession, obstructive uropathy, neurogenic dysfunction, intensified bacterial receptivity [23], and the compromised antibacterial environment in the prostate or vagina [24]. In this study, we found that febrile UTI was significantly related to the presence of upper tract UTI, bacteremia and septic shock, as was expected. However, although UTI patients with urosepsis, bacteremia or AKI tended to be older, with age-associated chronic diseases including hypertension, diabetes mellitus and congestive heart failure, it should be pointed out that febrile UTI patients were found to be relatively younger. This suggests that fever tends to be more frequently found in younger and better nourished UTI patients, likely reflecting the age- and frailty-dependent pyrogenic ability that may further affect clinical decision [25,26].

Acute UTI may result in the development of AKI, which is associated with a high morbidity and mortality rate [27,28,29]. As aforementioned, it is reasonable to believe that febrile UTI patients could be more prone to AKI as compared to afebrile ones, possibly mediated by septic shock or direct damage to the kidney. However, fever was found to be significantly less present in UTI patients suffering from AKI in our cohort, which was in clear contrast to the conditions of upper tract UTI, bacteremia and septic shock. Since multivariate logistic regression also reported the close relationships between AKI and upper tract UTI, bacteremia, as well as septic shock, this inverse association between fever and AKI was plausibly prominent in patients with acute cystitis without bacteremia. Of note, the underlying CKD was also found to be related to AKI in this cohort, as was expected. For instance, Hsu et al. demonstrated that patients with eGFR between 45 and 59 mL/min/1.73 m^2^ had an average twofold increase in adjusted OR of developing AKI as compared to subjects with eGFR of 60 mL/min/1.73 m^2^ or above [30]. Although renal impairment is known to be in close relationship with a lower febrile response as well [31,32,33], the mean difference between baseline eGFRs (70 versus 80 mL/min/1.73 m^2^) may not be sufficient to affect febrile status. However, whether the absence of fever may delay adequate therapy for UTI, thereby also contributing to complications such as AKI, remains to be elucidated.

Among the subgroup analysis of febrile UTI inpatients, we found that within all these febrile UTI inpatients, the absence of fever at ED was surprisingly associated with significantly longer hospital stay length as compared to patients with an initial presentation of fever at ED. Intriguingly, the absence of fever at ED was also significantly linked to younger ages, more frequent upper tract UTI, and more frequent subsequent AKI. These results pointed out that, other than the elderly, younger patients with UTI, especially those involving the upper tracts, more frequently presented with delayed fever, which may result in a delay of accurate diagnosis and adequate treatment that may further contribute to the significantly more frequent development of AKI. The fact that this “delayed” fever is more commonly observed in younger febrile UTI patients could be explained by their early awareness of the disease because of its symptoms. On the other hand, elderly patients are more likely to suffer from asymptomatic UTI, resulting in fever as one of their initial clinical presentations at ED [34]. If this is the case, it is even more important to point out that such timely arrivals to the ED of younger patients, who are assumed to have better outcomes, conversely had increased risks of subsequent AKI, indicating that the delayed clinical awareness in the absence of fever likely resulted in worse outcomes. Although the presence of initial fever may not seem reliable in distinguishing upper tract UTI or increased risk for AKI, the extent of the elevations in serum WBC and CRP levels did show predictive potential, despite the absence of fever. In short, our observational study pointed out the importance of avoiding complications, such as AKI, in UTI patients by enhancing clinical awareness of acute complicated UTI by putting more weight on leukocytosis and elevated CRP level, especially in younger patients with typical presentations, suggesting the involvement of either upper tract or systemic illness.

There are several limitations to this study. Firstly, our retrospective design may be prone to bias and missing data. To address these issues, we comprehensively collected the data using a standard form to reduce bias, and excluded patients with missing data. Second, although the treatments were prescribed by qualified physicians based on the standard treatment for UTI, medications, including aminoglycosides, non-steroid anti-inflammatory drugs, or contrast media, could also contribute to AKI, which may also cause bias. In general, nephrotoxic agents were avoided in patients with impaired renal function or wo were at risk of AKI, whereas contrast media may still be required in circumstances of complicated UTI, urosepsis or septic shock. Further prospective studies are warranted to confirm the relationship between nephrotoxic agents and AKI in UTI patients. Third, our results may also be affected by the multiple testing problem. In order to address this problem, we applied Holm–Bonferroni Correction in multivariate analysis, reporting both the *p*-values and each of their significance levels (α). Lastly, we could not apply the definition of AKI, based on urine output, of the Kidney Disease: Improving Global Outcomes (KDIGO) criteria in defining the development of AKI due to the lack of comprehensive urine output data. This could potentially cause an underestimation of the incidence of AKI in our UTI patients. Therefore, future prospective studies designed to record the urine output after admission along with serial serum creatinine measurements are also warranted to calculate the accurate incidence of AKI after UTI by applying the KDIGO criteria.

## 5. Conclusions

We consistently found that UTI patients with poorer baseline renal functions were more likely to be afebrile during their hospital stay, which was significantly linked to subsequent AKI. In addition, the absence of fever as an initial presentation at ED was also significantly related to the development of AKI, even in febrile UTI patients. The clinical awareness of upper tract UTI as well as the subsequent increased risk for AKI should be maintained in patients presenting with afebrile UTI, especially for younger patients with impaired baseline renal function or any UTI patients with substantially elevated serum WBC or CRP levels.

## Figures and Tables

**Table 1:** Characteristics of hospitalized patients with urinary tract infection with respect to fever.

Covariate	All (*n* = 1132)	Fever		*p*-Value
		Non (*n* = 407)	Yes (*n* = 725)	
Age (y/o)	66 ± 17	71 ± 16	64 ± 18	<0.001 **
Sex (male)	297 (26.2%)	121 (29.7%)	176 (24.3%)	0.045 ^¥^
Hospital stay length (days)	9 ± 5	9 ± 5	9 ± 5	0.794 *
Mean white blood cell (10^3^/μL)	13.32 ± 6.25	13.08 ± 6.62	13.46 ± 6.02	0.324 *
Platelets (10^3^/μL)	205.6 ± 110	212.7 ± 87.45	201.8 ± 120.7	0.111*
Hospitalized serum creatinine (mg/dL)	1.56 ± 1.57	1.86 ± 1.96	1.40 ± 1.27	<0.001 **
Baseline eGFR (mL/min/1.73 m^2^)	76.54 ± 29.90	70.37 ± 31.13	80.02 ± 28.64	<0.001 **
Diabetes mellitus	497 (43.9%)	188 (46.2%)	309 (42.6%)	0.245 ^¥^
Hypertension	614 (54.2%)	249 (61.2%)	365 (50.3%)	<0.001 ^¥^
Congestive heart failure	49 (4.3%)	20 (4.9%)	29 (4.0%)	0.468 ^¥^
Coronary artery disease	126 (11.1%)	53 (13%)	73 (10.1%)	0.130 ^¥^
Stroke	233 (20.6%)	118 (29%)	115 (15.9%)	<0.001 ^¥^
Indwelling Foley catheter	71 (6.3%)	41 (10.1%)	30 (4.1%)	<0.001 ^¥^
Upper urinary tract infection	510 (45.1%)	138 (33.9%)	372 (51.3%)	<0.001 ^¥^
Bacteremia	540 (47.7%)	127 (31.2%)	413 (57.0%)	<0.001 ^¥^
Urolithiasis	189 (16.7%)	61 (15.0%)	128 (17.7%)	0.248 ^¥^
Septic shock	218 (19.3%)	61 (15.0%)	157 (21.7%)	0.006 ^¥^
Acute kidney injury	159 (14.0%)	72 (17.7%)	87 (12.0%)	0.008 ^¥^
Multiple drug resistance isolate	391 (34.5%)	143 (35.1%)	248 (34.2%)	0.753 ^¥^
*Escherichia coli*	898 (79.3%)	294 (72.2%)	604 (83.3%)	<0.001 ^¥^
*Proteus mirabilis*	35 (3.1%)	18 (4.4%)	17 (2.3%)	0.053 ^¥^
*Klebsiella pneumoniae*	83 (7.3%)	35 (8.6%)	48 (6.6%)	0.220 ^¥^
*Enterococcus spp.*	35 (3.1%)	18 (4.4%)	17 (2.3%)	0.053 ^¥^
*Pseudomonas aeruginosa*	50 (4.4%)	23 (5.7%)	27 (3.7%)	0.130 ^¥^
Mortality	8 (0.7%)	4 (1.0%)	4 (0.6%)	0.468 ^¥¥^

*: Student *t*-test; **: Mann–Whitney U-test; ^¥^: Chi-Square test; ^¥¥^: Fisher’s exact test. eGFR: estimated glomerular filtration rate.

**Table 2:** Characteristics of hospitalized patients with urinary tract infection with respect to acute kidney injury.

Covariate	All (*n* = 1132)	Acute Kidney Injury		*p*-Value
		Non (*n* = 973)	Yes (*n* = 159)	
Age (y/o)	66 ± 17	65 ± 18	72 ± 12	<0.001 **
Sex (male)	297 (26.2%)	247 (25.4%)	50 (31.4%)	0.107 ^¥^
Hospital stay length (days)	9 ± 5	9 ± 4	13 ± 8	<0.001 **
Mean white blood cell (10^3^/μL)	13.32 ± 6.25	13.00 ± 5.82	15.35 ± 8.12	0.001 **
Platelets (10^3^/μL)	205.6 ± 110	207 ± 108	198.2 ± 121.4	0.005 **
Hospitalized serum creatinine (mg/dL)	1.56 ± 1.57	1.27 ± 1.23	3.32 ± 2.15	<0.001 **
Baseline eGFR (mL/min/1.73 m^2^)	76.54 ± 29.90	78.32 ± 30.00	65.70 ± 27.02	<0.001 **
Diabetes mellitus	497 (43.9%)	414 (42.5%)	83 (52.2%)	0.023 ^¥^
Hypertension	614 (54.2%)	502 (51.6%)	112 (70.4%)	<0.001 ^¥^
Congestive heart failure	49 (4.3%)	35 (3.6%)	14 (8.8%)	0.003 ^¥^
Coronary artery disease	126 (11.1%)	103 (10.6%)	23 (14.5%)	0.149 ^¥^
Stroke	233 (20.6%)	191 (19.6%)	42 (26.4%)	0.050 ^¥^
Indwelling Foley catheter	71 (6.3%)	57 (5.9%)	14 (8.8%)	0.155 ^¥^
Bacteremia	540 (47.7%)	441 (45.3%)	99 (62.3%)	<0.001 ^¥^
High Fever (≥38.4 °C)	725 (64.0%)	638 (65.6%)	87 (54.7%)	0.008 ^¥^
Urolithiasis	189 (16.7%)	135 (13.9%)	54 (34.0%)	<0.001 ^¥^
Septic shock	218 (19.3%)	146 (15.0%)	72 (45.3%)	<0.001 ^¥^
Multiple drug resistance isolate	391 (34.5%)	330 (33.9%)	61 (38.4%)	0.274 ^¥^
*Escherichia coli*	898 (79.3%)	786 (80.8%)	112 (70.4%)	0.003 ^¥^
*Proteus species*	35 (3.1%)	24 (2.5%)	11 (6.9%)	0.003 ^¥^
*Klebsiella species*	83 (7.3%)	66 (6.8%)	17 (10.7%)	0.080 ^¥^
*Enterococcus species.*	35 (3.1%)	27 (2.8%)	8 (5.0%)	0.137 ^¥^
*Pseudomonas species*	50 (4.4%)	44 (4.5%)	6 (3.8%)	0.670 ^¥^
Mortality	8 (0.7%)	5 (0.5%)	3 (1.9%)	0.089 ^¥¥^

**: Mann–Whitney U-test; ^¥^: Chi-Square test; ^¥¥^: Fisher’s exact test; eGFR: estimated glomerular filtration rate.

**Table 3:** Multivariate logistic regression analyses of factors related to acute kidney injury in patients with urinary tract infection.

Covariate	Acute Kidney Injury			
	*β*	OR (95% CI)	*p*-Value	Adjusted *α*
Age (y/o)	0.007	1.007 (0.992–1.023)	0.359	0.0050
Sex (male)	−0.097	0.908 (0.590–1.396)	0.659	0.0050
Mean white blood cell (10^3^/μL)	0.048	1.050 (1.020–1.080)	0.001	0.0033
Platelets (10^3^/μL)	0.000	1.000 (0.998–1.002)	0.740	0.0050
Baseline eGFR (mL/min/1.73 m^2^)	−0.009	0.991 (0.983–0.998)	0.010	0.0050
Diabetes mellitus	0.077	1.080 (0.732–1.593)	0.699	0.0050
Hypertension	0.620	1.859 (1.202–2.874)	0.005	0.0050
Congestive heart failure	0.625	1.869 (0.885–3.946)	0.101	0.0050
Coronary artery disease	−0.186	0.830 (0.470–1.467)	0.521	0.0050
Stroke	0.040	1.041 (0.663–1.634)	0.862	0.0050
Bacteremia	0.645	1.906 (1.262–2.878)	0.002	0.0042
High Fever (≥38.4 °C)	−0.653	0.521 (0.346–0.784)	0.002	0.0045
Urolithiasis	1.036	2.818 (1.845–4.303)	<0.001	0.0036
Septic shock	1.485	4.417 (2.948–6.618)	<0.001	0.0038
*Escherichia coli*	−0.393	0.675 (0.431–1.058)	0.086	0.0050

eGFR: estimated glomerular filtration rate. Adjusted significance level (α) was calculated by Holm–Bonferroni Correction.

**Table 4:** Characteristics of hospitalized patients with urinary tract infection with respect to bacteremia.

Covariate	All (*n* = 1132)	Bacteremia		*p*-Value
		Non (*n* = 592)	Yes (*n* = 540)	
Age (y/o)	66 ± 17	65 ± 19	68 ± 15	0.103 **
Sex (male)	297 (26.2%)	164 (27.7%)	133 (24.6%)	0.240 ^¥^
Hospital stay length (days)	99 ± 5	8 ± 4	10 ± 5	<0.001 **
Mean white blood cell (10^3^/μL)	13.32 ± 6.25	12.98 ± 5.71	13.70 ± 6.77	0.165 **
Platelets (10^3^/μL)	205.6 ± 110	220.9 ± 123.7	189.1 ± 89.9	<0.00 1 *
Hospitalized serum creatinine (mg/dL)	1.56 ± 1.57	1.47 ± 1.45	1.66 ± 1.68	0.041 *
Baseline eGFR (mL/min/1.73 m^2^)	76.54 ± 29.90	77.74 ± 31.53	75.24 ± 28.00	0.139 **
Diabetes mellitus	497 (43.9%)	228 (38.5%)	269 (49.8%)	0.001 ^¥^
Hypertension	614 (54.2%)	299 (50.5%)	315 (58.3%)	0.008 ^¥^
Congestive heart failure	49 (4.3%)	28 (4.7%)	21 (3.9%)	0.487 ^¥^
Coronary artery disease	126 (11.1%)	67 (11.3%)	59 (10.9%)	0.834 ^¥^
Stroke	233 (20.6%)	139 (23.5%)	94 (17.4%)	0.012 ^¥^
Indwelling Foley catheter	71 (6.3%)	44 (7.4%)	27 (5.0%)	0.092 ^¥^
High Fever (≥38.4 °C)	725 (64.0%)	312 (52.7%)	413 (76.5%)	<0.001 ^¥^
Urolithiasis	189 (16.7%)	79 (13.3%)	110 (20.4%)	0.002 ^¥^
Septic shock	218 (19.3%)	80 (13.5%)	138 (25.6%)	<0.001 ^¥^
Acute kidney injury	159 (14.0%)	60 (10.1%)	99 (18.3%)	<0.001 ^¥^
Multiple drug resistance isolate	391 (34.5%)	198 (33.4%)	193 (35.7%)	0.417 ^¥^
*Escherichia coli*	898 (79.3%)	442 (74.7%)	456 (84.4%)	<0.001 ^¥^
*Proteus species*	35 (3.1%)	21 (3.5%)	14 (2.6%)	0.354 ^¥^
*Klebsiella species*	83 (7.3%)	48 (8.1%)	35 (6.5%)	0.294 ^¥^
*Enterococcus species*	35 (3.1%)	23 (3.9%)	12 (2.2%)	0.106 ^¥^
*Pseudomonas species*	50 (4.4%)	41 (6.9%)	9 (1.7%)	<0.001 ^¥^
Mortality	8 (0.7%)	5 (0.8%)	3 (0.6%)	0.728 ^¥¥^

*: Student *t*-test; **: Mann–Whitney U-test; ^¥^: Chi-Square test; ^¥¥^: Fisher’s exact test; eGFR: estimated glomerular filtration rate.

**Table 5:** Multivariate logistic regression analyses of factors related to bacteremia in patients with urinary tract infection.

Covariate	Bacteremia			
	*β*	OR (95% CI)	*p*-Value	Adjusted *α*
Age (y/o)	0.012	1.012 (1.002–1.022)	0.016	0.0083
Platelets (10^3^/μL)	−0.003	0.997 (0.995–0.998)	<0.001	0.0042
Baseline eGFR (mL/min/1.73 m^2^)	0.000	1.000 (0.995–1.006)	0.878	0.0083
Diabetes mellitus	0.473	1.604 (1.223–2.104)	0.001	0.0045
Hypertension	0.210	1.234 (0.921–1.654)	0.159	0.0083
Stroke	−0.427	0.652 (0.465–0.915)	0.013	0.0083
Indwelling Foley catheter	−0.052	0.949 (0.545–1.653)	0.855	0.0083
High Fever (≥38.4 °C)	1.117	3.057 (2.306–4.052)	<0.001	0.0050
Urolithiasis	0.410	1.507 (1.06–2.143)	0.022	0.0083
Septic shock	0.557	1.746 (1.241–2.456)	0.001	0.0056
Acute kidney injury	0.606	1.832 (1.226–2.738)	0.003	0.0071
*Escherichia coli*	0.681	1.976 (1.414–2.76)	<0.001	0.0063

eGFR: estimated glomerular filtration rate. Adjusted significance level (α) was calculated by Holm–Bonferroni Correction.

**Table 6:** Characteristics of patients with febrile urinary tract infection during hospitalization, with or without fever in the emergency department.

Covariate	Fever (*n* = 714)	Non-Fever in Emergency Department (*n* = 210)	Fever in Emergency Department (*n* = 504)	*p*-Value
Age (y/o)	64 ± 18	62 ± 17	65 ± 18	0.028 *
Sex (male)	173 (24.2%)	55 (26.2%)	118 (23.4%)	0.430 ^¥^
Hospital stay length (days)	9 ± 5	10 ± 5	9 ± 4	0.002 **
Mean white blood cell (10^3^/μL)	13.44 ± 6.03	14.37 ± 6.41	13.06 ± 5.83	0.008 *
Platelets (10^3^/μL)	201.3 ± 120.9	213.2 ± 176.1	196.4 ± 88.0	0.089 *
Hospitalized serum creatinine (mg/dL)	1.38 ± 1.26	1.66 ± 1.70	1.27 ± 1.01	0.002 **
Baseline eGFR (mL/min/1.73 m^2^)	80.3 ± 28.66	78.88 ± 28.25	80.89 ± 28.84	0.393 *
C-reactive protein (mg/L) (*n* = 559)	11.24 ± 8.99	12.65 ± 9.78	10.61 ± 8.56	0.029 **
Diabetes mellitus	305 (42.7%)	90 (42.9%)	215 (42.7%)	0.961 ^¥^
Hypertension	362 (50.7%)	95 (45.2%)	267 (53%)	0.060 ^¥^
Congestive heart failure	29 (4.1%)	7 (3.3%)	22 (4.4%)	0.525 ^¥^
Coronary artery disease	71 (9.9%)	16 (7.6%)	55 (10.9%)	0.180 ^¥^
Stroke	115 (16.1%)	26 (12.4%)	89 (17.7%)	0.080 ^¥^
Indwelling Foley catheter	30 (4.2%)	10 (4.8%)	20 (4%)	0.630 ^¥^
Upper urinary tract infection	365 (51.1%)	125 (59.5%)	240 (47.6%)	0.004 ^¥^
Bacteremia	406 (56.9%)	112 (53.3%)	294 (58.3%)	0.219 ^¥^
Urolithiasis	123 (17.2%)	40 (19%)	83 (16.5%)	0.406 ^¥^
Septic shock	155 (21.7%)	50 (23.8%)	105 (20.8%)	0.379 ^¥^
Acute kidney injury	84 (11.8%)	37 (17.6%)	47 (9.3%)	0.001 ^¥^
Multiple drug resistance isolate	243 (34%)	74 (35.2%)	169 (33.5%)	0.661 ^¥^
*Escherichia coli*	597 (83.6%)	165 (78.6%)	432 (85.7%)	0.019 ^¥^
*Proteus mirabilis*	16 (2.2%)	9 (4.3%)	7 (1.4%)	0.025 ^¥^
*Klebsiella pneumoniae*	48 (6.7%)	14 (6.7%)	34 (6.7%)	0.969 ^¥^
*Enterococcus spp.*	15 (2.1%)	6 (2.9%)	9 (1.8%)	0.394 ^¥^
*Pseudomonas aeruginosa*	26 (3.6%)	12 (5.7%)	14 (2.8%)	0.056 ^¥^
Mortality	4 (0.6%)	3 (1.4%)	1 (0.2%)	0.079 ^¥¥^

*: Student T-test; **: Mann–Whitney U-test; ^¥^: Chi-Square test; ^¥¥^: Fisher’s exact test; eGFR: estimated glomerular filtration rate.

**Table 7:** Multivariate logistic regression model for factors related to fever during the stay in emergency department among patients with subsequent febrile urinary tract infections.

Covariate	Fever in ED			
	*β*	OR (95% CI)	*p*-Value	Adjusted *α*
Age (y/o)	0.017	1.017 (1.004–1.031)	0.010	0.0056
Baseline eGFR (mL/min/1.73 m^2^)	0.008	1.008 (1.001–1.016)	0.022	0.0056
Sex (male)	−0.125	0.883 (0.587–1.327)	0.549	0.0056
Hypertension	0.185	1.204 (0.823–1.760)	0.339	0.0056
Stroke	0.267	1.306 (0.776–2.198)	0.314	0.0056
Indwelling Foley catheter	−0.082	0.921 (0.394–2.153)	0.850	0.0056
Septic shock	−0.039	0.961 (0.630–1.468)	0.855	0.0056
Acute kidney injury	−0.834	0.434 (0.260–0.725)	0.001	0.0050
Bacteremia	0.195	1.216 (0.858–1.723)	0.272	0.0056
*Escherichia Coli*	0.455	1.577 (1.008–2.465)	0.046	0.0056

eGFR: estimated glomerular filtration rate. Adjusted significance level (α) was calculated by Holm–Bonferroni Correction.
